# Hyponatremia as the Presenting Feature of a Pituitary Abscess in a Calf

**DOI:** 10.3390/vetsci4010008

**Published:** 2017-02-06

**Authors:** Jamie L. Stewart, Maria C. Bates, B. Wade Edwards, Brian M. Aldridge

**Affiliations:** 1 Department of Veterinary Clinical Medicine, University of Illinois, Urbana, IL 61802, USA; ba311@illinois.edu; 2 Seventh Wave Laboratories, Maryland Heights, MO 63043, USA; mbates@7thwavelabs.com; 3 Department of Pathobiology, University of Illinois, Urbana, IL 61802, USA; bwdwrds2@illinois.edu

**Keywords:** cattle, endocrinopathy, pituitary, SIADH

## Abstract

A 2-month-old Simmental heifer presented for acute onset of neurological behavior. Laboratory tests confirmed the presence of hyponatremia, hypochloremia, and hypokalemia that improved with intravenous fluid therapy. Despite an initial cessation of neurological signs, symptoms re-emerged, and the heifer was euthanized due to poor prognosis. A pituitary abscess (*Trueperella pyogenes*) was observed on gross necropsy, suggesting that the effects of panhypopituitarism (inappropriate anti-diuretic hormone (ADH), adrenocorticotropic hormone (ACTH), and/or thyroid-stimulating hormone (TSH) secretion) may have resulted in the clinical findings. Pituitary abscess syndrome carries a poor prognosis due to the inability to penetrate the area with systemic antibiotic therapy. These findings highlight the unusual clinical presentations that may occur following pituitary abscess syndrome in cattle that practitioners need to consider when determining prognosis.

## 1. Presentation

A 2-month-old Simmental heifer presented to the University of Illinois Veterinary Teaching Hospital for a 2-week history of lethargy and an acute onset of star-gazing behavior that the owner noticed that morning. The owner reported that the heifer had a bout of diarrhea approximately 1 month ago that resolved quickly with treatment. On arrival to the hospital, the heifer was ambulatory, extremely agitated, running into objects, and displaying neurological behaviors such as star-gazing and head-pressing. Physical examination revealed an absent menace response, but a positive pupillary light response in both eyes. The heifer was tachycardic (108 beats/min, reference range: 40–100 beats/min [1]) and exhibited a normal respiratory rate (36 breaths/min, reference range: 25–40 breaths/min [1]). No abnormalities were appreciated on auscultation of the heart and lungs. Hydration status was adequate as determined by normal skin tenting and capillary refill time of <2 s, and rectal temperature was normal (38.7 °C, reference range: 38–39.3 °C [1]). Polioencephalomalacia was suspected, so the heifer was started on therapy consisting of thiamine (VetOne^®^, Boise, ID, USA) 20 mg/kg IM and dexamethasone (VetOne^®^, Boise, ID, USA) 0.1 mg/kg IM prior to obtaining bloodwork.

## 2. Diagnostics and Treatment

A Stat profile consisting of a blood gas and chemistry (Critical Care Xpress, Nova Biomedical, Waltham, MA, USA) was performed on heparinized whole blood, and revealed a respiratory alkalosis (pH 7.52, reference range: 7.35–7.50 [1]; and pCO_2_ 27.8 mmHg, reference range: 35–44 mmHg [1]) with a hyponatremia (116.2 mmol/L, reference range: 136–144 [1]), hypochloremia (93.8 mmol/L, reference range: 99–107 mmol/L [1]), hypokalemia (3.77 mmol/L, reference range: 3.8–5.2 mmol/L [1]), hyperglycemia (7.37 mmol/L, reference range: 2.2–5.6 mmol/L [1]), hyperlactatemia (7.1 mmol/L, reference range: 0.6–1.5 mmol/L [1]), and hypo-osmolality (236.7 mmol/L, reference range: 290–310 mmol/L [2]). Serum chemistry profile was consistent with these results, but also revealed a mildly elevated Gamma-Glutamyl Transferase (61 U/L, reference range: 6–17.4 U/L [1]). Complete blood cell count (leukogram and erythrogram) and fibrinogen were unremarkable. Urine was isosthenuric (1.010) and tested positive for hemolyzed blood using a urine dipstick (Urispec^®^ 11-Way, Henry Schein, Inc., New York, NY, USA).

Though the heifer had a history of diarrhea, there was no evidence of it at time of presentation. Additionally, physical examination revealed that she was of normal hydration (euvolemic), ruling out diarrhea as a cause of hyponatremia. Additionally, as the bloodwork also revealed a hypo-osmolality, the differential diagnoses were updated to include psychogenic polydipsia, syndrome of inappropriate anti-diuretic hormone secretion (SIADH), hypothyroidism, adrenal insufficiency, and nephrotic syndrome [2]. Nephrotic syndrome was placed lower on the list of differentials due to the absence of proteins found on the urine dipstick test. Advanced liver disease and renal failure were ruled out due to the presence of normal enzyme values on blood chemistry. A 16-gauge catheter was placed in the right jugular vein, and intravenous administration of 0.9% sodium chloride (Abbott Laboratories, Abbott Park, IL, USA) + 20 mEq/L KCl (Hospira, Lake Forest, IL, USA) at 550 mL/h (2× maintenance rate of 50 mL/kg/day) was initiated in an attempt to correct the hyponatremia at a rate of 12 mmol/L/day. Water buckets were removed from the stall due to suspected psychogenic polydipsia.

Serial blood gas and chemistry profiles were obtained to monitor and adjust fluid therapy, and are presented in Table 1. Glucose (4.6 mmol/L, reference range: 3.3–5.5 mmol/L [1]) and lactate (0.9 mmol/L, reference range: 0.6–1.5 mmol/L) levels were both resolved at 4 h after initiation of fluid therapy. The patient was switched to a balanced crystalloid solution (Plasma-Lyte, Abbott Laboratories, Abbott Park, IL, USA) with 10 mEq/L KCl added at 284 mL/h (maintenance rate of 50 mL/kg/day). On day 2, neurological behaviors had resolved, and the calf was exhibiting a normal suckle reflex.

On day 3, KCl supplementation was discontinued due to the normalization of both potassium and chloride levels (Table 1), but Plasma-Lyte administration was continued at 284 mL/h. Urine was submitted for urinalysis and was found to still be isosthenuric (SpGr 1.007) with a pH of 6.5 and contained trace glucose, rare white blood cells and epithelial cells, many bacteria, and moderate amorphous crystals. A urine clearance study was performed and ruled out underlying glomerular damage within the kidney (Table 2). Throughout the day, the heifer began to display intermittent neurologic signs, such as star-gazing, head-pressing, tremoring, teeth grinding, and ventral strabismus. Cerebral spinal fluid was collected from the lumbosacral space using aseptic technique, and cytological analysis revealed a severe nonseptic neutrophilic pleocytosis with a total protein of 1.8 g/L. The heifer was treated with florfenicol (NuFlor^®^, Merck Animal Health, Madison, NJ, USA) at 20 mg/kg IM for suspected meningitis or pituitary abscessation. By the morning of day 4, the heifer had continued to decline in mentation, becoming extremely dull and recumbent. Due to the declining condition and subsequent poor prognosis for recovery, euthanasia was elected.

## 3. Post-Mortem Findings

**Figure 1:**
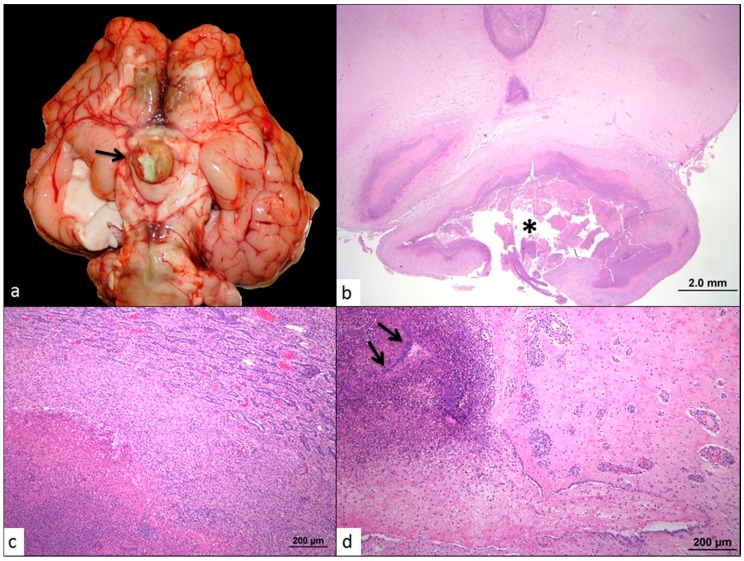
(**a**) Fresh brain, ventral surface: The pituitary contains a large amount of semi-solid, light yellow opaque material (arrow). Similar material extends along the meninges of the brainstem; (**b**–**d**) Hematoxylin and Eosin. (**b**) More than 90% of the pituitary gland is effaced and/or replaced by a large focus of inflammation (asterisk); (**c**) Compression of the pars distalis of the pituitary (upper right corner), by the pituitary abscess; (**d**) Inflammatory cells, necrotic debris, and colonies of coccobacilli (arrows) fill the ventricles and obscure portions of the ependymal lining and adjacent neuropil. Vessels in the adjacent neutrophil are cuffed by many inflammatory cells.

On necropsy, the pituitary gland was grossly enlarged, measuring approximately 2 cm in diameter (Figure 1a), and compressed the optic nerves. On cut section, the gland was cavitated and filled with a semi-solid, off-white to light yellow opaque exudate. The cerebral aqueduct and fourth ventricle were dilated and contained a small amount of similar off-white to yellow viscous exudate. The neuropil adjacent to the fourth ventricle was light yellow and gelatinous. Bilaterally, the lateral ventricles were dilated with compression of the adjacent cerebral neuropil and filled with abundant thin, colorless, transparent fluid. Multifocally throughout the cerebral cortical white matter were several pinpoint red foci. Histologically, more than 90% of the pituitary gland and portions of the adjacent hypothalamus were obscured and replaced by vast numbers of viable and necrotic neutrophils, abundant necrotic debris, fibrin, many macrophages, and myriad coccobacilli (Figure 1b). High numbers of epitheloid macrophages and moderate numbers of scattered lymphocytes and plasma cells were found surrounding the core inflammatory cell exudate and necrotic cellular debris. Additionally, many fibroblasts among a variably dense fibrous stroma were observed. The remaining portion of the pars distalis was compressed and arranged in short cords and tubules with large intervening bands of fibrous connective tissue and admixed neutrophils and fewer lymphocytes and plasma cells (Figure 1c). The inflammation extended into the adjacent neuropil and third ventricle with dilation of the ventricular lumen (Figure 1d). The large inflammatory infiltrate filled much of the lumen and obscured more than 75% of the ependymal cells. Throughout the adjacent hypothalamus, Virchow Robbin spaces were frequently expanded and contained high numbers of lymphocytes, plasma cells, and neutrophils. The meninges were also expanded and contained a similar inflammatory infiltrate and admixed edema, hemorrhage, and mixed bacteria. Bacterial culture of the pituitary abscess diagnosed the bacterial agent as *Trueperella pyogenes*.

## 4. Discussion

The diagnostic findings from this case suggested that the pituitary abscess was the primary cause of hyponatremia in this calf. Initially, psychogenic polydipsia, or “water intoxication” was the primary differential as has been previously described in cattle [4,5,6,7]. This condition was reported in calves given ad libitum access to water for the first time after weaning, resulting in excessive water intake without an appropriate renal response [7]. For water intoxication, hyponatremia, hypochloremia, and hemoglobinuria are commonly detected [7], consistent with the initial findings in the current case. However, water intake history was unknown in this calf, as it was housed with many other cattle. Treatment was initiated since signalment and diagnostic findings were consistent with this diagnosis, and reported cases responded well to fluid therapy. Clinical improvement was observed initially with normalized electrolyte parameters, and the decision was made to discontinue IV fluids.

While weaning from IV fluids, it became evident that intravenous fluid therapy was necessary to maintain normal electrolyte values, despite the calf not having free access to water. This finding raised concern about the true underlying cause of the hyponatremia. Though advanced renal failure was immediately ruled out due to the presence of normal BUN and creatinine values, nephrotic syndrome due to glomerular damage was still a possibility [2], although there are no reports of this occurring in cattle. Nephrotic syndrome was subsequently ruled out by the absence of protein in the urine and the normal urine electrolyte clearance test (Table 2 [3]). Nevertheless, neurological signs began to reappear despite the normalized electrolyte values, indicating that the inciting cause had not been corrected. The finding of a severe neutrophilic pleocytosis on cerebral spinal fluid analysis despite the lack of a systemic inflammatory response led to the suspicion of a pituitary abscess, which was later confirmed with necropsy.

A pre-mortem differential diagnosis was syndrome of inappropriate ADH secretion (SIADH). SIADH refers to the condition in which antidiuretic hormone (ADH) is secreted from the pituitary gland in the absence of normal osmotic stimuli, resulting in impaired water excretion [2]. The retention of water despite normal sodium excretion leads to a systemic dilution effect, evidenced by hyponatremia and hypo-osmolality, with resulting neurologic complications from acute edema of the brain cells [2]. While no edema was noticed on post-mortem evaluation of the brain, we speculate that it was either corrected pre-mortem with fluid therapy or that the neurological signs were a direct result of the meningitis detected. Conversely, a mild amount of diffuse pulmonary edema and congestion were reported at necropsy, likely another sequela to these systemic effects. To the authors’ knowledge, there are no reports in cattle that have implicated a pituitary abscess as a primary cause of SIADH, although it has previously been reported in humans [8,9] and a dog [10].

The post-mortem histological findings of almost complete effacement of the pituitary gland suggest a more likely clinical diagnosis of panhypopituitarism with secondary adrenal insufficiency. Adrenocorticotropic hormone (ACTH) is produced and secreted from the pituitary gland, and is responsible for stimulating cortisol production from the adrenal gland. In this condition, hyponatremia is caused by the lack of ADH inhibition from hypocortisolism [11]. This condition has been described in humans and is clinically similar to SIADH, but can be differentiated by testing adrenal function through serum cortisol measurements [11]. These cases were effectively managed with low-dose hydrocortisone administration [11]. Since only one dose of dexamethasone was administered in the current case, we cannot conclude that this was the primary pathological condition resulting from the pituitary gland destruction, though the pathology fits with the clinical picture. Thyroid-stimulating hormone (TSH) is another hormone produced and secreted from the pituitary gland, and is responsible for stimulating the production of thyroxine from the thyroid gland. Hypothyroidism has also been implicated in cases of euvolemic hyponatremia in humans, presumably due to decreased distal delivery of renal tubular fluid and nonosmotic stimulation of ADH secretion [2]. Though no diagnostics were performed to test for either of these conditions in the current case, it is likely that the pathology could have resulted from a combination of these effects. Diagnostics such as serum cortisol or thyroxine measurements could rule these disorders in or out [2,11]. However, as these tests are expensive and their results will not change the poor prognosis, they would not be practical to use in food animal practice.

In another similar case, a 1.7-year-old Simmental heifer presented with clinical signs that included depression, head-neck extension, persistently dropped jaw with impaired mastication, drooling, and dysphagia [12]. Similar to the current case, hypokalemia and hyponatremia were found on serum chemistry analysis [12]. However, the heifer also presented with a hyperglobulinemia, increased BUN and creatinine, and metabolic acidosis [12], which were not found in the current case. Pituitary abscess syndrome due to a sphenoid osteomyelitis was diagnosed on necropsy with *T. pyogenes* as the etiological agent [12]. Interestingly, despite isolation of the same bacterial agent and similar necropsy findings, the presentation and clinical findings were relatively different between this previous case and the current case. The degree of pituitary involvement could account for this, as it regulates several different functions within the body. Additionally, the pituitary abscess in the previous case was attributed to be secondary to a necrotizing purulent sinusitis of the right frontal sinus following dehorning [12]. In the current case, formation of a pituitary abscess was suspected to be a sequela from a previous systemic infection at a distant site (i.e., gastrointestinal), which has been previously described in cattle [13].

An interesting incidental finding on necropsy in this case was the presence of a moderate endometrial hyperplasia, which usually occurs in the presence of excess estrogen production [14]. In additional to secreting ADH, ACTH, and TSH, the pituitary gland also has an important role in regulating estrogen secretion via its production of follicle stimulating hormone and luteinizing hormone [14]. Since the calf was pre-pubertal, this finding is unusual and further supports a dysfunction of hormone secretion at the level of the pituitary gland associated with the pituitary abscess. In conclusion, this case demonstrates the unique presentation of a heifer calf with pituitary abscess syndrome. As discussed, this pathology could be a sequela of multiple hormone dysfunctions which would each need to be ruled out individually to determine if treatment could be attempted. However, as antibiotic penetration would be limited due to the abscess location, euthanasia would be the most practical option.

## Figures and Tables

**Table 1:** Serial bloodwork obtained for fluid therapy management.

Parameter	Reference Range [1]	Day 1, 5:00 PM	Day 1, 10:00 PM	Day 2, 8:00 AM	Day 2, 5:00 PM	Day 3, 9:00 AM	Day 3, 11:00 AM	Day 3, 4:00 PM
pH	7.35–7.45	7.50	7.45	7.55	7.47	N/A	7.62	7.45
Sodium	132–142 mmol/L	124.1	124.7	129.6	130.1	131	130.6	135.2
Chloride	95–106 mmol/L	93.8	94.0	96.3	97.8	89	109	99.4
Potassium	3.8–5.2 mmol/L	4.35	3.66	4.43	3.70	4.7	4.68	5.2
Osmolality	290–310 mmol/L	N/A	262.9	260.2	263.0	N/A	264.5	272.4

**Table 2:** Urine electrolyte clearance study performed to rule out underlying glomerular damage.

Test	Results	Reference Range (Mean) *
Creatinine, µmol/L	124	N/A
Calcium, mmol/L	2.4	2.2–2.4 (2.3)
Phosphorus, mmol/L	1.8	2.1–2.7 (2.4)
Sodium, mmol/L	131	138–144 (141)
Potassium, mmol/L	4.7	4.2–4.9 (4.5)
Chloride, mmol/L	89	98–102 (100)
Creatinine, Urine, µmol/L	4005	N/A
Calcium, Urine, mmol/L	2.9	0–2.3 (1.1)
Phosphorus, Urine, mmol/L	14	1.9–23 (12)
Sodium, Urine, mmol/L	68	28–162 (95)
Potassium, Urine, mmol/L	24	32–132 (82)
Chloride, Urine, mmol/L	69	37–187 (112)
Sodium % Clearance	1.61	0.68–3.26 (1.97)
Potassium % Clearance	15.8	33.1–65.5 (49.3)
Chloride % Clearance	2.41	1.3–5.0 (3.16)
Calcium % Clearance	3.71	0–3.17 (1.38)
Phosphorus % Clearance	23.7	0–32.3 (15.6)

* Derived from Neiger and Hagemoser [3].
